# Redetermination of the crystal structure of NbF_4_

**DOI:** 10.1107/S2056989016012081

**Published:** 2016-07-29

**Authors:** Jascha Bandemehr, Matthias Conrad, Florian Kraus

**Affiliations:** a Anorganische Chemie, Fachbereich Chemie, Philipps-Universität Marburg, Hans-Meerwein-Strasse 4, 35032 Marburg, Germany

**Keywords:** crystal structure, niobium, fluoride, SnF_4_ type

## Abstract

Single crystals of NbF_4_, niobium(IV) tetra­fluoride, were synthesized by disproportionation of Nb_2_F_5_ at 1273 K in a sealed niobium tube, extracted and studied by single-crystal X-ray diffraction. Previous reports on the crystal structure of NbF_4_ were based on X-ray powder diffraction data and the observed isotypicity to SnF_4_ [Gortsema & Didchenko (1965 ▸). *Inorg. Chem.* **4**, 182–186; Schäfer *et al.* (1965 ▸). *J. Less Common Met.* **9**, 95–104]. The data obtained from a single-crystal X-ray diffraction study meant the atomic coordinates could now be refined as well as their anisotropic displacement parameters, leading to a significant improvement of the structural model of NbF_4_. In the structure, the Nb atom is octahedron-like surrounded by six F atoms of which four are bridging to other NbF_6_ octa­hedra, leading to a layer structure extending parallel to the *ab* plane.

## Abstract 2

We present the first single-crystal X-ray structure analysis of NbF_4_ and compare some structural details with those obtained from previous powder X-ray diffraction studies. NbF_4_ crystallizes in the SnF_4_ structure type.

## Chemical context   

The first synthesis of niobium tetra­fluoride was reported by Schäfer and co-workers by reduction of niobium penta­fluoride with niobium metal (Schäfer *et al.*, 1964 ▸). According to Gortsema and coworker, a reduction of NbF_5_ with silicon is seemingly the best way to obtain pure NbF_4_ (Gortsema & Didchenko, 1965 ▸). The obtained products were reported as dark-blue or black powders, respectively (Gortsema & Didchenko, 1965 ▸, Schäfer *et al.*, 1964 ▸). However, we obtained green NbF_4_ single crystals among a green powder. NbF_4_ is moisture sensitive and deliquesces to a brown suspension. In aqueous medium a brown precipitate is formed. It is reported to be soluble in hydro­chloric acid, sulfuric acid or hydrogen fluoride (Schäfer *et al.*, 1965 ▸). The compound disproportionates under vacuum above 623 K to NbF_5_ and a fluoride of which the compositions were reported as NbF_2.37_ (Schäfer *et al.*, 1965 ▸) or NbF_3_ (Gortsema & Didchenko, 1965 ▸). In a sealed niobium ampoule NbF_4_ disproportionates at 825 K to NbF_5_ and Nb_2_F_5_ (Chassaing & Bizot, 1980 ▸). Infrared spectra (Dickson, 1969 ▸), UV/Vis-spectra (Chassaing & Bizot, 1980 ▸) and powder X-ray patterns are available for NbF_4_ (Gortsema & Didchenko, 1965 ▸, Schäfer *et al.*, 1965 ▸). Magnetic measurements show that NbF_4_ orders anti­ferromagnetic in contrast to the other niobium tetra­halides which are reported to be diamagnetic (Chassaing & Bizot, 1980 ▸).

## Structural commentary   

The lattice parameters obtained by our single-crystal structure determination of *a* = 4.0876 (5), *c* = 8.1351 (19) Å are in good agreement with those obtained previously from powder X-ray diffraction data recorded on film (*a* = 4.081, *c* = 8.162 Å; Gortsema & Didchenko, 1965 ▸; *a* = 4.08 (3), *c* = 8.16 (1) Å; Schäfer *et al.*, 1965 ▸).

**Figure 1:**
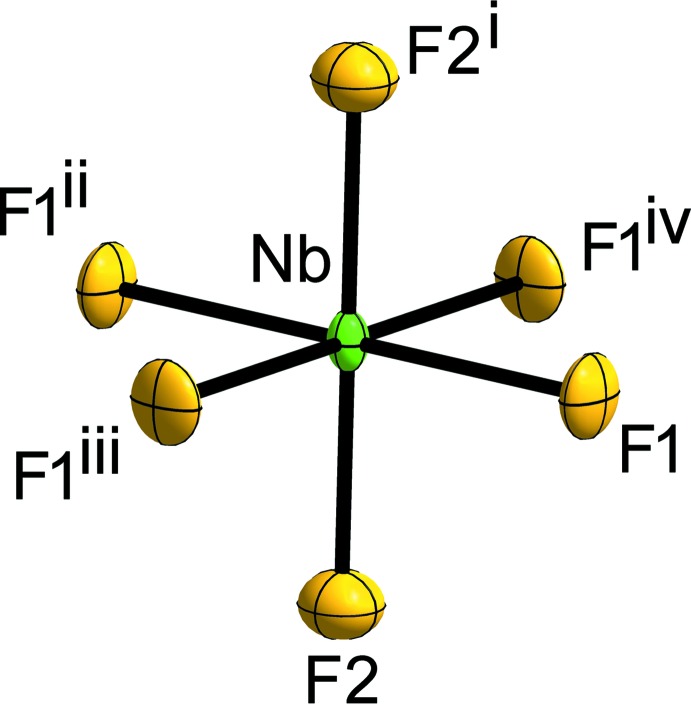
A section of the crystal structure of the title compound displaying the coordination polyhedron around the Nb atom. Displacement ellipsoids are shown at the 70% probability level at 293 K. [Symmetry codes: (i) −*x*, −*y*, −*z*; (ii) *x*, *y* − 1, *z*; (iii) −*y*, *x*, *z*; (iv) −*y* + 1, *x*, *z*.]

**Figure 2:**
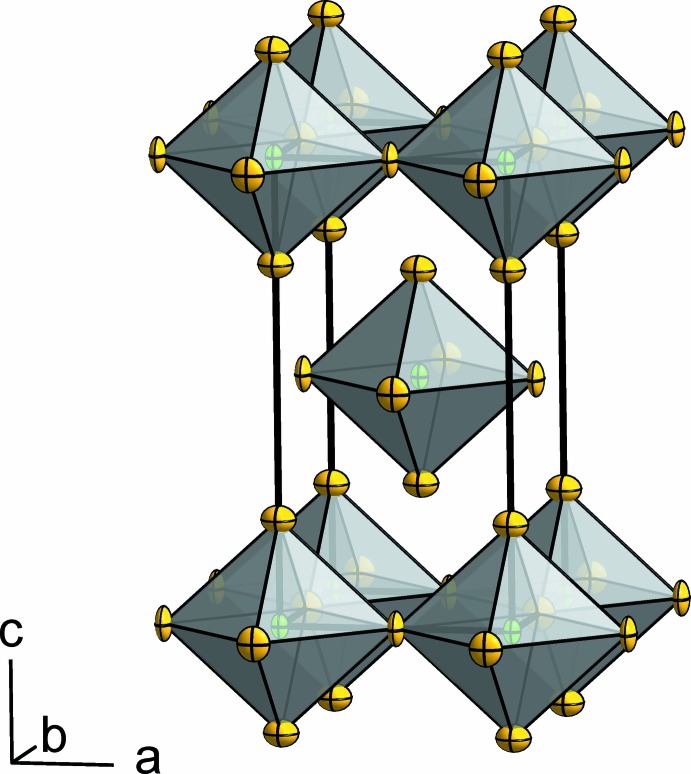
The crystal structure of NbF_4_ presented as a polyhedron model. Displacement ellipsoids are shown at 70% probability level at 293 K.

NbF_4_ crystallizes in the SnF_4_ structure type (Hoppe & Dähne, 1962 ▸; Bork & Hoppe, 1996 ▸), which has been discussed extensively and its structural relationship to the NaCl structure type (Müller, 2013 ▸) deduced. The Nb atom resides on Wyckoff position 2*a* (site symmetry 4/*mmm*) and is octahedron-like coordinated by six fluorine atoms of which four are bridging to further octa­hedra, thus corner-sharing connections are obtained. These Nb—(μ-F) distances, with the F1 atoms residing on the 4*c* (*mmm*.) position, are observed to be 2.0438 (3) Å and the Nb—F—Nb angle is 180° due to space-group symmetry. The structure models based on powder diffraction data yielded 2.041 (Gortsema & Didchenko, 1965 ▸) and 2.042 Å (Schäfer *et al.*, 1965 ▸) for these Nb—F distances. The Nb—(μ-F) distance is similar to the respective ones of NbF_5_ [2.06 (2) and 2.07 (2) Å; Edwards, 1964 ▸] but shorter than the respective one of Nb_2_F_5_ [2.1179 (4) Å; Knoll *et al.*, 2006 ▸]. Two fluorine atoms (F2, 4*e*, 4*mm*) of the title compound are not bridging and are *trans* arranged at the Nb atom. As expected, the non-bridging F2 atoms show shorter Nb—F distances of 1.8524 (19) Å; these values differ significantly from those of 2.0405 (Gortsema & Didchenko, 1965 ▸) and 2.040 Å (Schäfer *et al.*, 1965 ▸). The F2 atoms are surrounded by twelve F atoms (eight symmetry-equivalent F1 and four F2 atoms) in the shape of a distorted cubocta­hedron. A ‘central’ F2 atom is displaced by 0.24 Å from the center of this cubocta­hedron towards the Nb atom to which it is bound. Hence the expected deviation from *m* 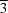 *m* (*O*_h_) to 4/*mmm* (*D*_4h_) symmetry is much more obvious. In comparison to the Nb—F distances (non-bridging F-atoms) of NbF_5_, which are reported to be 1.75 (5) and 1.78 (5) Å (Edwards, 1964 ▸), an elongation is observed. This is attributed to the higher oxidation state of the Nb atom in NbF_5_. Fig. 1 ▸ shows a section of the crystal structure displaying the coordination polyhedron around the Nb atom. As in SnF_4_, infinite layers with Niggli formula ^2^_∞_[NbF_4/2_F_2/1_] are present and extend parallel to the *ab* plane. The crystal structure is shown in Fig. 2 ▸.

## Synthesis and crystallization   

Niobium tetra­fluoride was synthesized by heating brown Nb_2_F_5_ (54,4 mg, 0,16 mmol) to 1273 K in a sealed niobium tube (22 mm, 4 mm i.d., 6 mm o.d.) which was placed upright in an evacuated sealed silica tube. The heating rate was 20 K h^−1^ and the maximum temperature was held for two days. The niobium ampoule had been charged under nitro­gen atmosphere in a glove box and sealed by arc welding. Nb_2_F_5_ was also synthesized in a niobium ampoule (33 mm, 4 mm i.d., 6 mm o.d.) starting from niobium metal and niobium penta­fluoride with a heating rate of 16 K h^−1^. The maximum temperature of 1073 K was held for two days. The ampoules were allowed to cool to room temperature and were opened under inert atmosphere. A powder X-ray diffraction pattern of the green product shows the reflections of NbF_4_, Nb and an yet unidentified phase. It seems that Nb_2_F_5_ disproportionates to NbF_5_ and Nb, and by cooling NbF_4_ is formed. This assumption is supported by the observation that high pressure inside the ampoule blew it up. The pressure is likely induced by gaseous NbF_5_, and the disproportionation of Nb_2_F_5_ to Nb and NbF_5_ is known from the literature (Schäfer *et al.*, 1965 ▸). A selected single crystal of NbF_4_ was investigated using X-ray diffraction and diffraction data measured at room temperature.

## Refinement   

As a starting model for the structure refinement, the atomic coordinates of the SnF_4_ structure type were used. Crystal data, data collection and structure refinement details are summarized in Table 1 ▸. One reflection (112) was omitted from the refinement as it was affected by the primary beam stop.

## Supplementary Material

Crystal structure: contains datablock(s) I. DOI: 10.1107/S2056989016012081/wm5309sup1.cif

Structure factors: contains datablock(s) I. DOI: 10.1107/S2056989016012081/wm5309Isup2.hkl

CCDC reference: 1496052

Additional supporting information: crystallographic information; 3D view; checkCIF report

## Figures and Tables

**Table 1:** Experimental details

Crystal data	
Chemical formula	NbF_4_
*M*_r_	168.91
Crystal system, space group	Tetragonal, *I*4/*m* *m* *m*
Temperature (K)	293
*a*, *c* (Å)	4.0876 (5), 8.1351 (19)
*V* (Å^3^)	135.93 (5)
*Z*	2
Radiation type	Mo *K*α
μ (mm^−1^)	4.32
Crystal size (mm)	0.06 × 0.04 × 0.01

Data collection	
Diffractometer	Stoe IPDS 2T
Absorption correction	Integration (*X-RED32* and *X-SHAPE*; Stoe & Cie, 2009 ▸)
*T*_min_, *T*_max_	0.664, 0.925
No. of measured, independent and observed [*I* > 2σ(*I*)] reflections	1392, 167, 167
*R*_int_	0.057
(sin θ/λ)_max_ (Å^−1^)	0.944

Refinement	
*R*[*F* ^2^ > 2σ(*F* ^2^)], *wR*(*F* ^2^), *S*	0.014, 0.032, 0.98
No. of reflections	167
No. of parameters	10
Δρ_max_, Δρ_min_ (e Å^−3^)	0.69, −0.58

Computer programs: *X-AREA* (Stoe & Cie, 2011 ▸), *X-RED32* (Stoe & Cie, 2009 ▸), *SHELXL2014* (Sheldrick, 2015 ▸) and *DIAMOND* (Brandenburg, 2015 ▸).

## supplementary crystallographic information

### Crystal data

**Table:**

NbF_4_	*D*_x_ = 4.127 Mg m^−^^3^
*M**_r_* = 168.91	Mo *K*α radiation, λ = 0.71073 Å
Tetragonal, *I*4/*m**m**m*	Cell parameters from 2534 reflections
*a* = 4.0876 (5) Å	θ = 5.0–42.2°
*c* = 8.1351 (19) Å	µ = 4.32 mm^−^^1^
*V* = 135.93 (5) Å^3^	*T* = 293 K
*Z* = 2	Plate, green
*F*(000) = 154	0.06 × 0.04 × 0.01 mm

### Data collection

**Table:**

Stoe IPDS 2T diffractometer	167 independent reflections
Radiation source: sealed X-ray tube, 12 x 0.4 mm long-fine focus	167 reflections with *I* > 2σ(*I*)
Plane graphite monochromator	*R*_int_ = 0.057
Detector resolution: 6.67 pixels mm^-1^	θ_max_ = 42.1°, θ_min_ = 5.0°
rotation method scans	*h* = −7→5
Absorption correction: integration (*X-RED32* and *X-SHAPE*; Stoe & Cie, 2009)	*k* = −7→7
*T*_min_ = 0.664, *T*_max_ = 0.925	*l* = −14→15
1392 measured reflections	

### Refinement

**Table:**

Refinement on *F*^2^	Primary atom site location: isomorphous structure methods
Least-squares matrix: full	Secondary atom site location: isomorphous structure methods
*R*[*F*^2^ > 2σ(*F*^2^)] = 0.014	*w* = 1/[σ^2^(*F*_o_^2^) + (0.025*P*)^2^] where *P* = (*F*_o_^2^ + 2*F*_c_^2^)/3
*wR*(*F*^2^) = 0.032	(Δ/σ)_max_ < 0.001
*S* = 0.98	Δρ_max_ = 0.69 e Å^−^^3^
167 reflections	Δρ_min_ = −0.58 e Å^−^^3^
10 parameters	Extinction correction: SHELXL2014 (Sheldrick, 2015), Fc^*^=kFc[1+0.001xFc^2^λ^3^/sin(2θ)]^-1/4^
0 restraints	Extinction coefficient: 0.026 (5)

### Special details

**Table:**

Geometry. All esds (except the esd in the dihedral angle between two l.s. planes) are estimated using the full covariance matrix. The cell esds are taken into account individually in the estimation of esds in distances, angles and torsion angles; correlations between esds in cell parameters are only used when they are defined by crystal symmetry. An approximate (isotropic) treatment of cell esds is used for estimating esds involving l.s. planes.

### Fractional atomic coordinates and isotropic or equivalent isotropic displacement parameters (Å^2^)

**Table:**

	*x*	*y*	*z*	*U*_iso_*/*U*_eq_	
Nb	0.0000	0.0000	0.0000	0.00798 (9)	
F1	0.0000	0.5000	0.0000	0.0167 (3)	
F2	0.0000	0.0000	0.2277 (2)	0.0209 (3)	

### Atomic displacement parameters (Å^2^)

**Table:**

	*U*^11^	*U*^22^	*U*^33^	*U*^12^	*U*^13^	*U*^23^
Nb	0.00583 (10)	0.00583 (10)	0.01230 (11)	0.000	0.000	0.000
F1	0.0211 (7)	0.0056 (5)	0.0235 (6)	0.000	0.000	0.000
F2	0.0239 (5)	0.0239 (5)	0.0149 (5)	0.000	0.000	0.000

### Geometric parameters (Å, º)

**Table:**

Nb—F2^i^	1.8524 (19)	Nb—F1^iii^	2.0438 (3)
Nb—F2	1.8524 (19)	Nb—F1^iv^	2.0438 (3)
Nb—F1	2.0438 (3)	F1—Nb^v^	2.0438 (3)
Nb—F1^ii^	2.0438 (3)		
			
F2^i^—Nb—F2	180.0	F1—Nb—F1^iii^	90.0
F2^i^—Nb—F1	90.0	F1^ii^—Nb—F1^iii^	90.0
F2—Nb—F1	90.0	F2^i^—Nb—F1^iv^	90.0
F2^i^—Nb—F1^ii^	90.0	F2—Nb—F1^iv^	90.0
F2—Nb—F1^ii^	90.0	F1—Nb—F1^iv^	90.0
F1—Nb—F1^ii^	180.0	F1^ii^—Nb—F1^iv^	90.0
F2^i^—Nb—F1^iii^	90.0	F1^iii^—Nb—F1^iv^	180.0
F2—Nb—F1^iii^	90.0	Nb^v^—F1—Nb	180.0

Symmetry codes: (i) −*x*, −*y*, −*z*; (ii) *x*, *y*−1, *z*; (iii) −*y*, *x*, *z*; (iv) −*y*+1, *x*, *z*; (v) *x*, *y*+1, *z*.
